# Depth Data Denoising in Optical Laser Based Sensors for Metal Sheet Flatness Measurement: A Deep Learning Approach

**DOI:** 10.3390/s21217024

**Published:** 2021-10-23

**Authors:** Marcos Alonso, Daniel Maestro, Alberto Izaguirre, Imanol Andonegui, Manuel Graña

**Affiliations:** 1 Robotics and Automation Group, Electronic and Computer Science Department, Faculty of Engineering, Mondragon University, Loramendi Kalea, 4, 20500 Arrasate-Mondragon, Spain; dmaestro@mondragon.edu (D.M.); aizagirre@mondragon.edu (A.I.); iandonegui@mondragon.edu (I.A.); 2 Computational Intelligence Group, CCIA Department, UPV/EHU, Paseo Manuel de Lardizabal 1, 20018 San Sebastian, Spain; manuel.grana@ehu.es

**Keywords:** laser triangulation, metal sheet flatness measurement, smooth surface reconstruction, depth data denoising, Convolutional Neural Networks, deep learning, residual learning

## Abstract

Surface flatness assessment is necessary for quality control of metal sheets manufactured from steel coils by roll leveling and cutting. Mechanical-contact-based flatness sensors are being replaced by modern laser-based optical sensors that deliver accurate and dense reconstruction of metal sheet surfaces for flatness index computation. However, the surface range images captured by these optical sensors are corrupted by very specific kinds of noise due to vibrations caused by mechanical processes like degreasing, cleaning, polishing, shearing, and transporting roll systems. Therefore, high-quality flatness optical measurement systems strongly depend on the quality of image denoising methods applied to extract the true surface height image. This paper presents a deep learning architecture for removing these specific kinds of noise from the range images obtained by a laser based range sensor installed in a rolling and shearing line, in order to allow accurate flatness measurements from the clean range images. The proposed convolutional blind residual denoising network (CBRDNet) is composed of a noise estimation module and a noise removal module implemented by specific adaptation of semantic convolutional neural networks. The CBRDNet is validated on both synthetic and real noisy range image data that exhibit the most critical kinds of noise that arise throughout the metal sheet production process. Real data were obtained from a single laser line triangulation flatness sensor installed in a roll leveling and cut to length line. Computational experiments over both synthetic and real datasets clearly demonstrate that CBRDNet achieves superior performance in comparison to traditional 1D and 2D filtering methods, and state-of-the-art CNN-based denoising techniques. The experimental validation results show a reduction in error than can be up to 15% relative to solutions based on traditional 1D and 2D filtering methods and between 10% and 3% relative to the other deep learning denoising architectures recently reported in the literature.

## 1. Introduction

Increasingly stringent specifications in terms of flatness and surface quality in the manufacture of sheet metal products are becoming more demanding of real-time 100% quality-control processes. The end customer expects not only excellent mechanical and processing properties but also a high long-term value and a high quality of modern metallic materials. To meet these high expectations, the steel industry needs intelligent quality-control systems endowed with high-precision in-line sensors for real-time measurements.

In the manufacture of parts and assemblies, especially when parts are required to be assembled over a surface, flatness is a critical specification requirement. Any flatness defect will cause an undesirable optical effect and impact the overall appearance of the assembly. This need for zero defect manufacturing arises in areas as varied as the manufacture of stainless steel sheets used in professional kitchens, metal panels for exterior decoration in architectural projects, or the manufacture of aluminum sheets in the automotive industry. For this reason, it is highly desirable to carry out a quality control in real time during the metal sheet manufacturing covering 100% of its surface in order to ensure that the required industry quality standards are met.

With the advances in computer vision technology, optical flatness sensors have became widespread [1] allowing manufacturing line human operators to measure manifest flatness, i.e., flatness not hidden by tension, at high line speeds, thus enabling real-time monitoring as well as a high degree of automation in the production phase. Most optical surface flatness inspection systems used in the metal sheet industry are based on the laser triangulation principle [2,3,4].

The large real-time inspection capabilities of these optical sensors are impeded by the non-linear high-frequency fluctuations induced in the steel sheet surface by the mechanical processes that take place in the manufacturing line, the juddering of the metal strip due to forward traction, as well as the shearing processes that cut to length the sheet. Under these circumstances, achieving a highly accurate flatness measurement requires a high performance signal denoising method to be applied to the height profile captured by the 3D sensor, removing the noise corresponding to such non-linear high-frequency fluctuations. The literature [5,6,7,8,9,10,11] presents different sensors based on laser triangulation, requiring the use of two or more laser lines to filter out external noise sources and reconstruct an accurate and smooth continuous 3D map of the metal sheet surface.

The main contribution of this paper is a novel deep learning architecture for the reconstruction of the range image captured by the 3D sensor removing the high-frequency noise due to mechanical processes in order to allow accurate flatness measurements for quality control. This deep learning architecture is inspired in the U-Net [12], originally developed for semantic segmentation. Instead of returning as output an decomposition of the image into regions, our architecture returns the noise-free range image by using a noise estimation module. The architecture is validated against synthetic and real range images that exhibit the most significant noise modalities produced by the mechanical processing induced vibrations on the steel sheet surface. Real data have been collected from an industrial roll leveling and cut-to-length line where the developed 3D sensor is installed. Moreover, the architecture is compared against image denoising deep learning architectures reported in the literature. To this end, we have retrained these architectures with our data from scratch.

The remainder of this paper is organized as follows: Section 2 reviews the industrial context regarding techniques and devices used to measure metal sheets flatness. Section 3 describes our noise model for the generation of synthetic data. Section 4 reviews computational approaches for image denoising, setting the stage for our proposal. Section 5 and Section 6 present the proposed deep learning architecture for range image denoising and the collected Dataset, respectively. Section 7 reports the experimental results. Finally, Section 8 gives our conclusions and directions of future work.

## 2. Industrial Context

In order to inspect rolled products achieving accurate measurements and classification of flatness defects, it is necessary to capture the geometry of the steel sheet as it moves through the processing line. With sheet feeding rates reaching speeds of up to 120 m/min, real-time inspection imposes very strict requirements for accurate surface flatness quality control. The most typical flatness defects are wavy edges, centre buckles, and bow defects, which appear as low-frequency variations in the metal strip surface height.

On account of the strict requirements for real time quality control of surface flatness, the time efficiency of noise filtering methods poses a major challenge. Most of the literature [7,9,11] addresses this problem relying on the use of traditional filtering methods or explicit noise modeling, requiring extensive fine-tuning to adequately adapt to different noise levels, struggling in preserving details, and leading to local (sensor-specific) solutions. Several successful applications of machine learning and fuzzy systems modeling for the detection of surface defects in flat steel products can be found in the literature [13,14,15], but they do not extend to the categorization of flatness defects. There are even machine learning approaches to link different types of defects with their causes [16,17].

Contrary to traditional hand crafted filtering methods, Convolutional Neural Networks (CNNs) are tuned by automated learning techniques guided by error minimization carried out by stochastic gradient descent and backpropagation algorithm. They have improved sensor data interpretation, analysis and control algorithms, being capable of dealing with non-linearities, noise, and uncertainty. In this regard, CNNs have become the state-of-the-art machine learning approach in many applications [18,19,20,21,22]. Recently, CNNs have been applied to classify surface defects in cold-rolled strips [23], and flatness measure prediction [24] from measurements of contact sensors attached to the roll mill instead of optical or range images of the surface. In order to adapt their 1D data from the sensor readings they fold these vectors into small images (5 × 8 or 20 × 20) which are the input for the CNNs, following the convention that CNNs are image classifiers or regressors. Note that the goal in [24] is the prediction of an overall measure of flatness from linear sensor readings.

However, to the best of the authors knowledge, there are no studies yet on CNN or other deep-learning-based methods to filter data obtained from optical flatness sensors in order to accurately reconstruct the surface of metal strips. In this regard, we are specifically interested in assessing the denoising performance of deep learning architectures when the input range image data contain high levels of non-linear noise.

### Actual Sensor Installation

**Figure 1:**
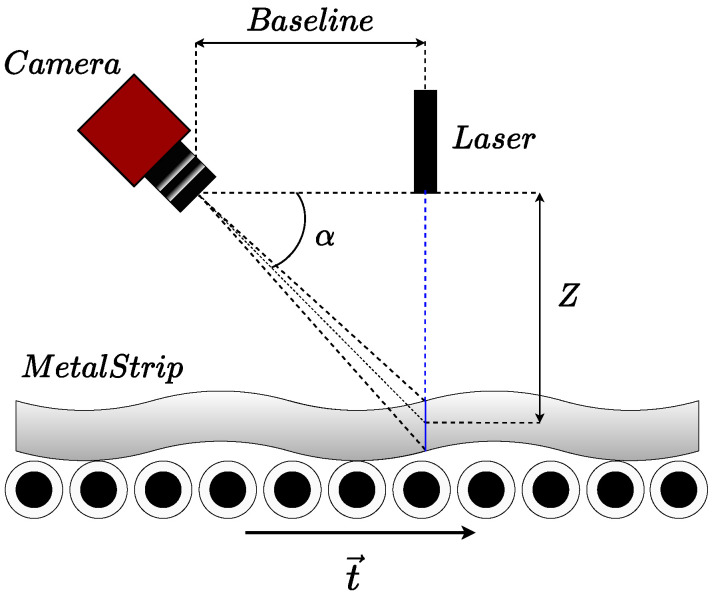
The flatness sensor scheme used for data acquisition, consisting of a single laser line laser triangulation scheme.

The flatness data were acquired with a simplified version of the optical flatness sensor described in [10]. The flatness sensor is comprised of a single illuminating linear laser source perpendicular to the metal sheet translation axis and a CCD camera capturing the area illuminated by the laser. In this simplified sensor version, the baseline separation between camera and the laser source is $\Delta_{B}=900\;$ mm, and the triangulation angle is $\alpha =45^{\circ }$ so that the center of the camera captures the middle of the laser line at Z=0 mm. The laser line emitter is collimated, and its wavelength is λ=450 nm, while its line aperture is $90^{\circ }$. The camera features a 2048 × 2048 matrix CCD sensor, and the focal length of the lens is f=6 mm, placed at Z=1140 mm over a moving steel strip. Figure 1 shows the scheme of the sensor.

**Figure 2:**
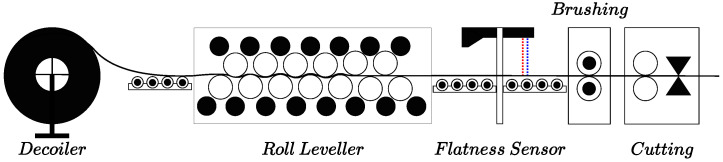
The experimental production line scheme and the optical flatness sensor placement. Blue and Red lines refer to laser planes used for pseudo-groundtruth calculation in real data experiments. Blue line refers to the laser plane which is further used for training, validation, and testing the proposed CBRDNet.

Figure 2 shows the scheme of the production line and the placement of the optical flatness sensor. Steel coils which are reduced to a specific thicknesses by rolling and annealing and wound into a roll. These steel coils are further processed in a roll leveling and shearing line where they are cut to length. The range sensor was placed before the cutting tool, so the steel sheet surface propagates the vibrations induced by the cutting shocks. Each type of steel coil possesses different mechanical properties and thickness. As a result, they exhibit different propagation responses to the vibrations induced in the metal sheet during the leveling and cutting processes. This fact adds variability and robustness requirements to the proposed network.

## 3. Noise Model for Synthetic Data Generation

Generating physically consistent surface data are crucial to train the proposed CBRDNet and increase its denoising generalization capability. However, modeling such metal surfaces is impeded by the lack of accurate experimental data. Custom metrology devices, such as coordinate measuring machines (CMM), rely on static measuring conditions and, thus, fail to retrieve the most characteristic surface deformation caused by the tensile and trachle stresses occurring at the metal strip roll leveling and cut to length processes. To cope with this lack of data, our synthetic samples rely on a model of experimentally reconstructed surface data shown in [10], which reproduce the most common defects in a roll leveler processing line, as well as the coupling noise produced by mechanical elements, such as cutting stage.

**Figure 3:**
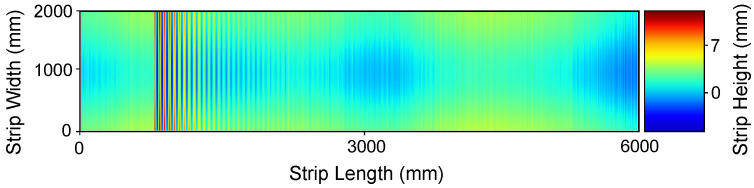
Synthetic flatness sensor data. (Color Online).

We model the range image captured by our sensor from metal surface data by a function that combines a high-frequency and high-amplitude bump produced by the cutting stage, modeled as a local Gaussian signal ψ(x,y), a superposition of a set of stationary waves φ(x,y), a low-frequency carrier θ(y) and a Gaussian noise term ρ(x,y) modeling the data acquisition electronics error,

(1) S(x,y)=φ(x,y)+ψ(x,y)+θ(y)+ρ(x,y)

where

(2) $$\begin{array}{c} \varphi \left(x,y\right)=\sum_{n=0}^{N}\sum_{m=0}^{M}\alpha_{n,m}cos\left(\frac{2\pi nx}{\lambda_{x}}\right)cos\left(\frac{2\pi my}{\lambda_{y}}\right)\\ +\sum_{n=0}^{N}\sum_{m=0}^{M}\beta_{n,m}cos\left(\frac{2\pi nx}{\lambda_{x}}\right)sin\left(\frac{2\pi my}{\lambda_{y}}\right)\\ +\sum_{n=0}^{N}\sum_{m=0}^{M}\gamma_{n,m}sin\left(\frac{2\pi nx}{\lambda_{x}}\right)cos\left(\frac{2\pi my}{\lambda_{y}}\right)\\ +\sum_{n=0}^{N}\sum_{m=0}^{M}\delta_{n,m}sin\left(\frac{2\pi nx}{\lambda_{x}}\right)sin\left(\frac{2\pi my}{\lambda_{y}}\right) \end{array}$$

is a real-valued 2D Fourier series, where the amplitudes α=δ=[0,5] and β=γ=0. $\lambda_{x}=\lambda_{y}=\left[0,0.1\right]$ are the wavelengths in the *x* and *y* directions,

(3) $$\psi \left(x,y\right)=\frac{sin(f_{b}yA_{b})}{1+4{\left[\frac{\left(y-y_{0}\right)}{L_{b}}\right]}^{2}}$$

is a high-frequency, high-amplitude Gaussian wave mixed with a low-frequency carrier modeling the bump produced by the cutting device, where $f_{b}=5$ represents the bump carrier frequency, $A_{b}=\left[1,3\right]$ stands for the bump amplitude, $L_{b}=\left[10,20\right]$ is the bump wave attenuation, and

(4) $$\theta \left(y\right)=A_{c}cos\left(K_{c}y\right)$$

is a low-frequency carrier that sets the offset of the surface data along the transversal y-direction, where $A_{c}=\left[0,0.5\right]$ is the carrier amplitude and $K_{c}=\left[0,0.1\right]$ represents the frequency in the *y* direction. Finally, ρ(x,y) is the electronic noise that arises during data acquisition caused by the discrete nature of radiation, i.e., the fact that the optical sensor captures an image by collecting photons. Considering some assumptions, this noise can be approximated by an additive model in which the noise has a zero-mean Gaussian distribution determined by its variance $\sigma_{n}^{2}=\left[0.1,0.35\right]$. That is, each value in the noisy data is the sum of the real value and a random, Gaussian distributed noise value. The defined intervals of variation and constant values for these variables have been selected in order to obtain synthetic data that are as close as possible to that acquired by the sensor in real experiments. We disregarded strict boundary conditions, such as Dirichlet conditions due to the free form nature of the unrolled metal coils on the machine. A synthetic surface generated using this model is shown in Figure 3.

As shown in Figure 3, the proposed noise model allows us to generate synthetic data that are very similar to that acquired by the sensor in real experiments. The degree of concordance between our model and experimental data have been qualitatively validated by visual inspection. We cannot tune the model quantitatively because the noise source is not observable. We cannot observe the noise separated from the actual metal sheet surface, and the wave propagation and dumping properties are dependent of the actual metal sheet mechanical properties. We postulate that the success of the denoising system trained on the synthetic data are indirect proof of the validity of the model.

## 4. Deep Learning Denoising Approaches

An autoencoder is an unsupervised neural network architecture that is trained to reproduce the input as its output. It has a typical structure as a pair of funnels attached by the short end. The first funnel compresses the input data into a lower-dimension encoding, while the second funnel decompresses the encoding trying to recover the original input data. The encoder seeks to obtain a robust latent representation of the original data, which is often used for other purposes, such as features for another classification module. Autoencoders have been a popular field of study in neural networks in recent decades. The first applications of this type of neural networks date back to the 1980s [25,26,27]. Autoencoders have been used for classification, clustering, anomaly detection, dimensionality reduction, and signal denoising [28].

Proposed by Vincent et al. [29], the Denoising Autoencoders (DAEs) are an extension of classic autoencoders where the model is taught to predict original uncorrupted data from corrupted input data, i.e., the decoder attempts to reconstruct a clean version of the corrupted input from the autoencoder latent representation.

The encoder function *f* takes an input $\tilde{x}$ and maps it to a hidden representation y computed as:

(5) $$y=f_{\theta }\left(\tilde{x}\right)=h\left(W\tilde{x}+b\right)$$

where *h* is a typically nonlinear transfer function, W and b are the encoder network parameters, and θ=(W,b).

The output x, having a similar form to $\tilde{x}$, is reconstructed from y by the decoder *g*

(6) $$x=g_{\theta^{\prime }}\left(y\right)=h^{\prime }\left(W^{\prime }y+b^{\prime }\right)$$

where $h^{\prime }$ is similar to h, $W^{\prime }$ and $b^{\prime }$ are the decoder network parameters, and $\theta^{\prime }=\left(W^{\prime },b^{\prime }\right)$.

The DAE training procedure consists on learning the parameters W, $W^{\prime }$, b, and $b^{\prime }$ that minimise the autoencoder reconstruction error between the groundtruth x and the reconstruction $g_{\theta^{\prime }}\left(f_{\theta }\left(\tilde{x}\right)\right)$, using a suitable cost function. Typically, the function is minimised using Stochastic Gradient Descent (SGD) [30] for small batches of corrupted and clean sample pairs.

Convolutional Denoising Autoencoders (CDAEs) are Denoising Autoencoders implemented using convolutional encoding and decoding layers. Because CDAEs use CNNs for extracting high-order features from images, CDAEs differ from standard DAEs in that their parameters are shared across all input image patches to maintain spatial locality. Different studies show that CDAEs achieve better image processing performance when compared to standard DAEs [31,32].

The U-Net [12] has a encoding–decoding architecture inspired in the autoencoder with skip connections [33] that transfer the data from the encoder layers to the decoding layers. Input–output pairs are images and their desired semantic pixel labelling providing segmentation of the image in one shot. It has shown exceptional results for image segmentation and image restoration tasks [34,35,36]. Depending on the architectural modifications made to U-Net, it can be used to achieve different tasks beyond segmentation. Isola et al. [37] used U-Net as a generator to perform image-to-image translation tasks such as in the case of aerial images and their correspondence in maps or the conversion of gray-scale images to color images through adversarial learning. Jansson et al. [38] investigated the use of U-Net as a voice separator, using the magnitude of the spectrogram of the audio containing the mix of different singing voices as the input. Zhang et al. [39] modified U-Net with a residual block and proposed it as a tool for extracting roads from aerial maps.

State-of-the-art 2D deep learning image denoising methods that will be compared with our proposal are CBDNet [40], NERNet [41], BRDNet [42], FFDNet [43], and CDnCNN_B [44]. CBDNet is a convolutional blind denoising network [40] that is composed of a noise estimation module and a non-blind denoising module that accepts the noise estimation to compute the clean image. The noise estimation module is a CNN without pooling (i.e., no dimension reduction), while the denoising module is a U-shaped network as discussed above. The work reported in [40] uses a realistic noise model that includes in-camera processing to generate synthetic images with known noise component for network training. The noise estimation and removal network NERNet [41] inherits the two module structure of CBDNet. The noise estimation module is enriched with a pyramidal feature fusion block that provides multi-scale noise estimation, while the CNN components are dilated convolutions. The noise removal module is U-shaped using dense convolution and dilation selective blocks. The synthetic images were generated adding white Gaussian noise (AWGN). In the batch renormalization denoising network BRDNet [42], the batch renormalization is claimed to address the internal covariate shift and small mini-batch problems. The network is composed of upper and lower networks. Upper network is composed of residual learning modules with batch renormalization, while the lower network includes also dilated convolution blocks. Contrary to the previous networks, no explicit noise estimation module is designed. Noise is assumed to be AWGN. The fast and flexible denoising network FFDNet [43] is also designed for cleaning AWGN corrupted images. FFDNet is a CNN whose inputs are downsampled subimages and a noise level map, it does not have a module to estimate the noise. The denoising convolutional neural network (DnCNNs) [44] is able to handle Gaussian denoising with unknown noise level. The DnCNN uses residual learning in order to estimate the noise component of the image, which is later removed from the noisy image to obtain the clean image.

## 5. Proposed Deep Learning Image Denoising Architecture

We apply of U-Net architecture as a generalized denoising method for surface reconstruction from noisy range images. The proposed network should be capable of denoising the degraded range images as an alternative to traditional image denoising techniques like spatial filtering, transform domain filtering, or wavelet thresholding methods [45]. A denoising method should remove high- and low-frequency noises, reconstructing the original surface. Results presented in the literature show that CNNs outperform traditional techniques for denoising tasks [46,47]. Furthermore, once trained, CNNs are computationally very efficient as they may be run on high-performance graphic processing units (GPUs) [48,49].

Our study proposes a convolutional blind residual denoising network model (CBRDNet) based on the U-Net architecture for denoising flatness sensor data. Since in real-world scenarios only noisy input data are provided, correct estimation of the noise level has proven to be challenging [40]. Therefore, incorporating a noise estimation block, can enhance the network generalization capabilities as shown by Lan et al. [50] and Guo et al. [41]. Besides that, the combination of both synthetic and real noisy data in the model training is expected to improve the network’s denoising efficiency [51].

The structure and denoising functionality of the proposed network are described within the following sub-section.

### 5.1. Network Architecture

**Figure 4:**
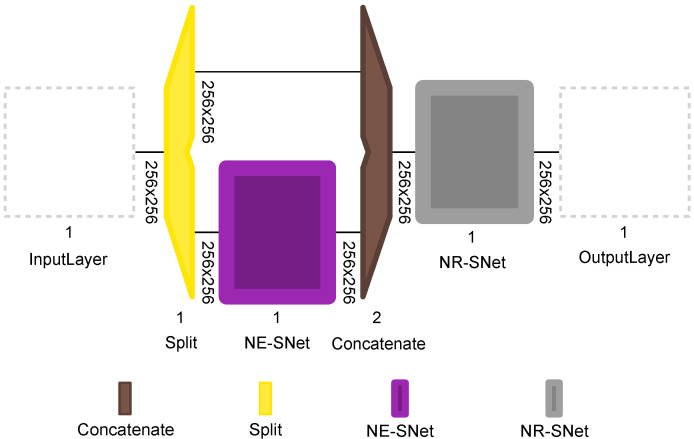
Overall scheme of the proposed CBRDNet network for close to real-time flatness data denoising.

The proposed CBRDNet architecture consists of mainly two stages, a blind residual noise estimation subnetwork (NE-SNet) and a noise removal subnetwork (NR-SNet). The overall scheme of the proposed network is shown in Figure 4

**Figure 5:**
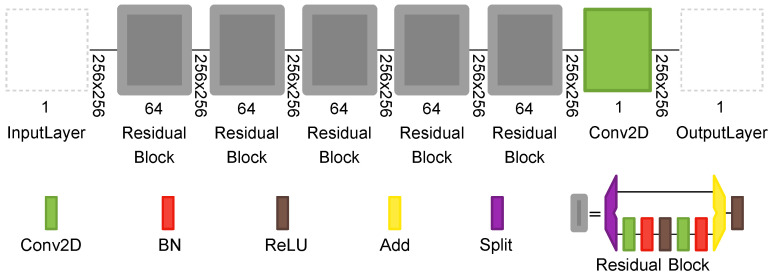
The proposed Noise Estimation Subnetwork NE-SNet composed of residual learning blocks.

The NE-SNet subnetwork takes a noisy observation and produces an estimated noise level map. It is composed of residual learning blocks that were first proposed as part of the ResNet architecture [52]. The layers of this subnetwork will increasingly separate image structure from noise, creating a noise map that will be used later in the denoising stage. The NE-SNet is composed of five residual blocks with no pooling, each of which has two convolutional (Conv2D) layers with Batch Normalization (BN) and Rectified Linear Unit (ReLU) layers. The number of feature channels in each Conv2D layer is set to 64, and the filter size is set to 3×3. The scheme of the NE-SNet subnetwork is shown in Figure 5.

**Figure 6:**
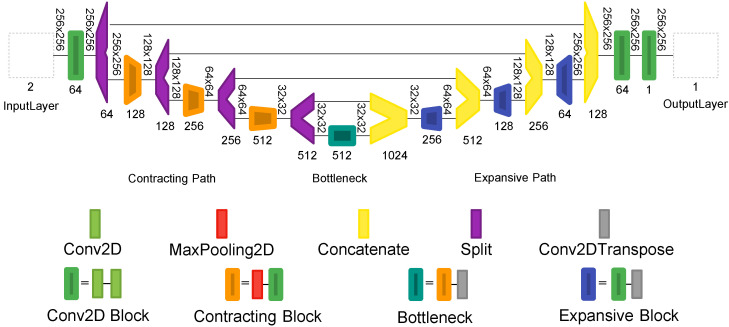
The proposed Noise Removal Subnetwork NR-SNet following a U-net architecture.

The NR-SNet subnetwork is based on a traditional U-Net. This subnetwork is divided into two major paths: contracting (encoder) and expanding (decoder). The contracting path is comprised of downsampling layers consisting of a MaxPooling2D layer and two Conv2D layers with a filter size of 3×3 and “same“ padding configuration. Each contracting block halves the size of feature maps and doubles the number of feature channels, starting with 64 channels in the first stage and ending with 512 channels in the last. The bottleneck connects both the expanding path and the contracting path; herein, the data has been resized to 32×32×512. Similarly, the expanding path also comprises four upsampling blocks, which are composed of two Conv2D layers followed by a Conv2D Transpose. Each expanding block doubles the size of feature maps and halves the number of feature channels. We used concatenation layers to merge the feature maps in the expanding path with the corresponding feature maps in the contracting path. The last layer is a 1×1 Conv2D. The original U-Net architecture for image segmentation uses a sigmoid activation function in this last layer. Instead, our proposed architecture uses a linear activation function in order to recover the denoised image. The scheme of the NR-SNet subnetwork is given in Figure 6.

### 5.2. Training the Model

Given a 3D dataset encompassing data recovered from the laser based optical flatness sensor and synthetic 3D data described in Section 6, we generate a set of depth images, which are decomposed into patches for processing. Using this dataset of local patches, we train our network to reconstruct the denoised versions of input depth images. In order to train the CBRDNet, we use the ADAM [53] algorithm with β=0.9. Following most CNN-based data denoising methods, our network adopts the mean squared error (MSE) as the loss function and the initialization strategy of He [54]. The mini-batch size is 10, and each patch size is 256×256 pixels. The mini-batch size has been selected as a trade-off between our limited computational capabilities and the desired network generalization performance. Experimental results demonstrate that small batch sizes with small learning rates result in more reliable and stable training, better generalization performance, and a much lower memory footprint [55,56]. The model is trained for 100 epochs, with the learning rate for the first 20 epochs set to $10^{e-3}$ and the learning rate $10^{e-4}$ used to fine-tune the model. These settings are the same for all experiments discussed in this paper for uniformity. Besides that, both ReLU and LeakyReLu [57] have been tested as output layer activation functions in the CBRDNet training, the obtained results were almost identical and are shown in Section 7. We trained all the networks in this paper on a single NVIDIA^®^ Geforce^®^ RTX 2080 Super GPU with an on-board frame buffer memory of 8GB GDDR6, 3072 CUDA^®^ Cores operating at 1815 MHz, compute capability 7.5, and Turing Generation microarchitecture, CUDA^®^ 10.1 and CUDNN 7.6.1). The machine is equipped with an Intel^®^ Core i9-10900K CPU @ 3.70GHz processor with 10 cores and 32 GB of RAM.

## 6. Dataset

The dataset used for both training and testing of the proposed architecture is composed of real production line and synthetic range image samples of steel coils from a roll levelling and shearing line. The synthetic data are used as a kind of data augmentation aiming to improve the network denoising performance because of the difficulties faced collecting a real dataset comprising a wide range of representative samples. Additionally, in real-world measurements the metal sheet is not free from tensile stresses during the manufacturing processes causing its elongation. After cutting the metal strip in single smaller sheets, the tensile stress release results in surface deformations. Thus, measurements obtained by an offline precision measuring device like a coordinate measuring machine (CMM) cannot be used as a validation ground truth for online measurement methods, whereas synthetic samples do.

In this paper, we generate 5500 synthetic noisy data samples using the noise model described in Section 3 together with 5500 real noisy samples from six different coils which are described in Section 6.1. The dataset is divided into a training set (80%), a validation set (10%) and a test set (10%).

### 6.1. Real Production Line Data

The experimental data coming from the real production line consists of 5500 samples from six different steel coils.

The specifications of the six steel coils are as follows: Two S235JR coils, a carbon (non-alloy) steel formulated for primary forming into wrought products with thicknesses of 3 mm, 8 mm and 1200 mm width, respectively, Young modulus E=205 GPa, Poisson ratio μ=0.301, yield stress σ=215 MPa, annealed and skin passed. One S420ML coil, a special structural steel with a thickness of 7 mm and 2000 mm width, Young modulus E=190 GPa, Poisson ratio μ=0.29, yield stress σ=410 MPa, it is an iron alloy steel manufactured by rolling. One S355M coil, an alloy steel formulated for primary forming into wrought products with a thickness of 3 mm and 1500 mm width, Young modulus E=190 GPa, Poisson ratio μ=0.29, yield stress σ=360 MPa, a middle carbon steel manufactured by rolling, annealing and skin passing. Two S500MC coils, a hot-rolled, high-strength low-alloy (HSLA) with excellent engineering bending and cutting characteristics with a thickness of 3 mm, 6 mm and 2200 mm width, respectively, Young modulus E=210 GPa, Poisson ratio μ=0.304, yield stress σ=500 MPa, produced through thermomechanical rolling. A summary is given in Table 1.

**Figure 7:**
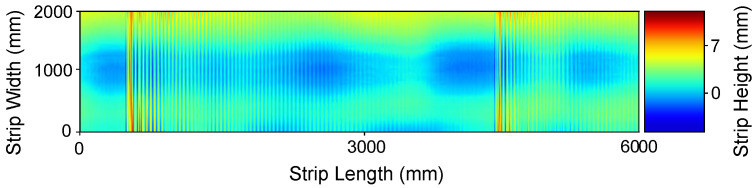
Raw optical flatness sensor data. (Color online).

The coils are roughly 800 m long. In each measurement cycle, the optical flatness system senses 9000 mm. High-amplitude disruptive noises from the cutting station, as well as the mechanical processes carried out during the manufacturing greatly contaminate the flatness information generating noisy ripples on the metal strip sensor data. Additionally, the conveyor system generates high-frequency waves as a result of the metal strip advance. This interference patterns result in a complex spatial waveform, causing flatness information and surface defects difficult to detect. A raw depth data sample from one of these steel coils, captured by the optical flatness sensor, is visualised in Figure 7.

## 7. Results

In this section, we assess the proposed CBRDNet for denoising both synthetic sheet samples and real data from the 3D flatness sensor. The proposed denoising network is employed to reconstruct both simulated and real data in order to test its ability to remove non-linear noises caused by mechanical manipulation of the metal sheet during the manufacturing process.

The metal sheet’s flatness corresponds to its levelness when it is tension free. The I-Unit [58] is widely used as the standardized measurement unit of flatness. For the I-Unit calculation in a metal sheet with a sinusoidal surface, a series of virtual lines are drawn to model the surface profile. The I-Unit is computed over them and the reported flatness is the average over all lines. For this reason we compare our 2D methods with 1D denoising methods. We recall that the aim of the present work is to provide a CNN-based denoising method to be be applied to range images obtained by optical sensors installed in metal sheet leveling and shearing production lines. The denoised surface range data will be used to carry out the necessary flatness measurement. Accordingly, the results provided below compare the denoised synthetic sheet samples and real ones with its corresponding groundtruth. The error measurements are expressed in millimeters.

### 7.1. Synthetic Data Results

**Figure 8:**
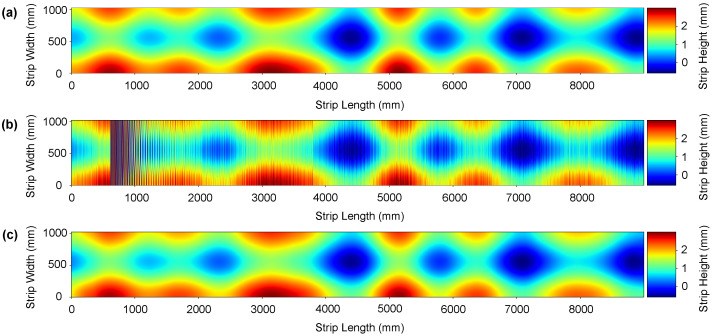
An instance of the denoising result on a synthetic strip. (**a**) Depicts the noise-free ground truth surface, (**b**) shows the noise corrupted surface, and (**c**) represents the denoised surface reconstructed using the proposed network. (Color online).

**Figure 9:**
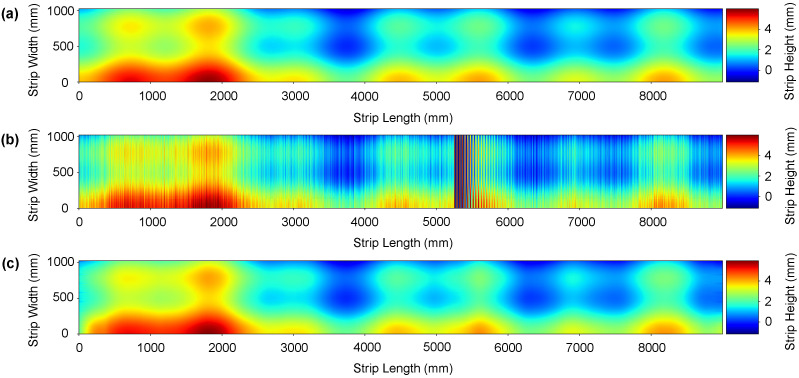
An instance of the denoising result on a synthetic strip. (**a**) Depicts the noise-free ground truth surface, (**b**) shows the noise corrupted surface, and (**c**) represents the denoised surface reconstructed using the proposed network. (Color online).

We conducted three different comparative analyses. First, we apply some traditional 1D filtering methods such as Moving Average, Butterworth IIR [59,60], Savitzky-Golay FIR [61,62], Chebyshev Type II [63], and piecewise cubic Hermite interpolation [10] filters. Secondly, we apply 2D wavelet-based denoising methods. Specifically, we compute results using Daubechies, Symlets, Meyer, Coiflets, and Fejer-Korovkin wavelets [64,65,66]. Finally, we compare the performance of CBRDNet against some state-of-art 2D deep learning image denosing methods, specifically NERNet [41], CBDNet [40], BRDNet [42], FFDNet [43], and CDnCNN_B [44]. Instances of synthetic data denoising results are shown in Figure 8 and Figure 9, where (a) is the noise-free sample, (b) is the noisy surface data and, finally, (c) is the denoised surface estimated using our method.

For the comparative analysis with traditional 1D filtering methods we divided the resulting metal sheet surface in virtual longitudinal strips, also called fibers [58,67]. For each fiber, we applied the following 1D denoising approaches:

A Butterworth IIR filter. This filter provides the optimum balance of attenuation and phase response. It has no rippling effect in the passband or stopband, and as a result, it is frequently referred to as a maximally flat filter. The Butterworth filter provides flatness at the cost of a somewhat broad transition area from passband to stopband, with typical transitory characteristics. It has the following characteristics: a smooth monotonic response (no ripple), it has the slowest roll-off for equivalent order filters, and a more linear passband phase response than other methods. A Butterworth IIR third-order digital filter with a cutoff frequency of 6 dB below the passband value of 0.01 specified in normalized frequency units is used.

A Savitzky-Golay FIR smoothing filter, which is a variation of the FIR average filter that can effectively retain the targeted signal’s high-frequency content while still not eliminating as much noise as a FIR average. Savitzky-Golay filters maintain various moment orders better than other smoothing approaches, which generally retain peak widths and heights. It has the following characteristics: a computation time proportional to window width, it preserves the area, position and width of peaks, and flattens peaks less than moving average with same window width. A third-order Savitzky-Golay FIR smoothing filter with a frame length of 99 samples is used in our experiments.

A Moving Average filter was also applied, which is a method used to smooth data by calculating a series of averages of different subsets of the entire dataset. It is a form of finite impulse response filter with the following characteristics: an optimal approach for reducing random noise while retaining a sharp step response, in general term is a good smoother filter, conceptually it is the simplest to implement, but on the contrary has a poor low-pass filter (frequency domain) and a slow roll-off and terrible stopband attenuation characteristics. A moving-average filter with a 33-sample-long sliding window is used for the comparison experiments.

A Chebyshev Type II filter has been applied. This filter is also known as an inverse filter, it does not roll off and has no ripple in the passband, but it has equiripple in the stopband. The main characteristics of this filter are: it is maximally flat in the passband and has a faster roll-off than Butterworth but slower roll-off than Chebyshev Type I. We used a third-order low-pass Chebyshev Type II filter with a stopband attenuation of 33 dB and a stopband edge frequency of 0.02 specified in normalised frequency units.

Finally, a piecewise cubic Hermite interpolation filter has been used. This filter uses both the height surface information and its derivative calculated from a dual laser sensor data series. It is continuous in shape and its derivative. In comparison to the Savitzky–Golay, Butterworth, Chebyshev, and Average Mean filters used for surface reconstruction in [10], this method achieved a 41 percent improvement.

Because we have the ground truth surface, we can compute the error of our denoising process. Table 2 shows the comparative results of the denoising approaches described above when applied to the synthetic surface. MAE improvements achieved by our method range from three times better when compared to the Hermite filtering approach to 6 times better when compared to the Chebyshev filter approach. Similar improvements are achieved in term of RMSE.

**Figure 10:**
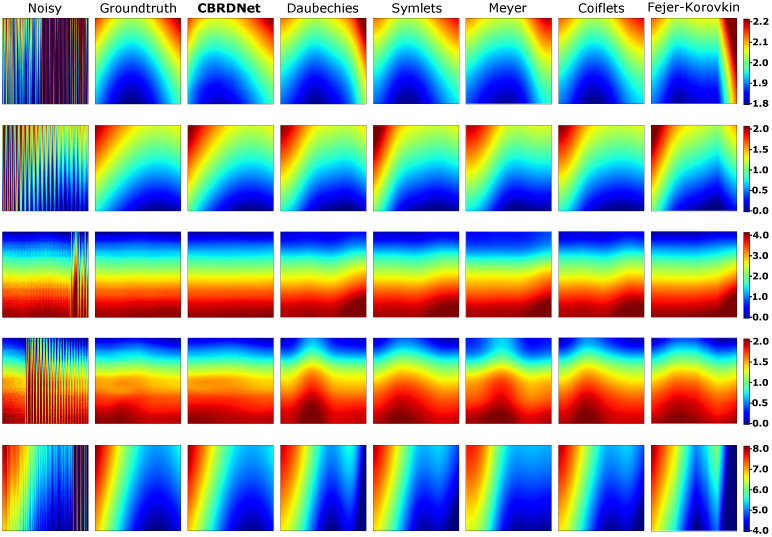
Comparison of our proposed CBRDNet with 2D wavelet-based denoising techniques. Noisy synthetic samples have very low SNR, hence the the groundtruth surface of the samples is almost lostt in some samples. To facilitate the comparison with the denoised samples, the color scale of the images corresponding to the first column, i.e., noisy sample, is clipped. Color scale values are expressed in millimeters (mm). (Color online).

In addition, we conducted 2D wavelet-based denoising methods. The number of vanishing moments *N* and the denoising threshold are the metaparameters for this approach. According to the current research, disregarding the computational cost of the wavelet transform (WT), higher vanishing moments would yield better performance [68,69]. We selected the following wavelets: Daubechies (dbN), N=4, Symlets (symN) N=8, Meyer (dmey), Coiflets (coifN), N=4, and Fejer-Korovkin (fkN), N=4. We performed the WT of data samples up to 8 levels. For denoising, wavelet transform coefficients below an empirically selected WT threshold are set to zero. An inverse wavelet transform is used after that to transform the processed signal back to the original spatial domain. Because the wavelet coefficients are affected by values outside the extent of the signal under consideration, to avoid boundary effects, the first and last 4 samples were removed in the processed input data. Table 2 shows the comparative results. MAE improvements achieved by our method range from 2.5 times better when compared to the Fejer-Korovkin filtering approach to 1.3 times better when compared to the Symlets filter approach. Similar improvements are achieved in term of RMSE. For a graphical representation of these results, we provide the denoising results on five data samples in Figure 10.

**Figure 11:**
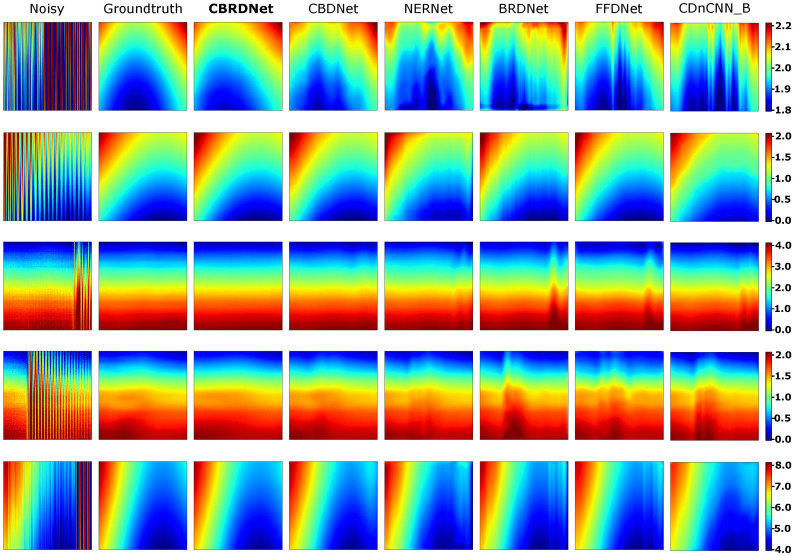
Comparison of our proposed CBRDNet with other methods on five different samples. Note that because of the low SNR, the geometrical surface of the samples is hidden by the induced perturbations. To facilitate the comparison with the denoised samples, the color scale of the images corresponding to the first column, i.e., noisy sample, is clipped. Color scale values are expressed in millimeters (mm). (Color online).

Finally, we compared the architecture presented in this article to the five earlier stated CNN-based approaches. Comparing various deep learning algorithms is a challenging task because of the large number of hyperparameters that must be appropriately tuned during the network training process. Notwithstanding, the aforementioned architectures were trained and assessed 100 times on the same dataset to obtain the statistical results listed in Table 2. Furthermore, for a clearer graphical representation of denoising performance, we provide the outcomes of these methods on five data samples, see Figure 11. When compared to the groundtruth the CBRDNet results are very close to the real ones, MAE improvements range from 2.5 times better when compared to CDnCNN_B and 1.2 times better when compared to CBDNet. Similar improvements are measured in terms of RMSE.

### 7.2. Real Data Results

**Figure 12:**
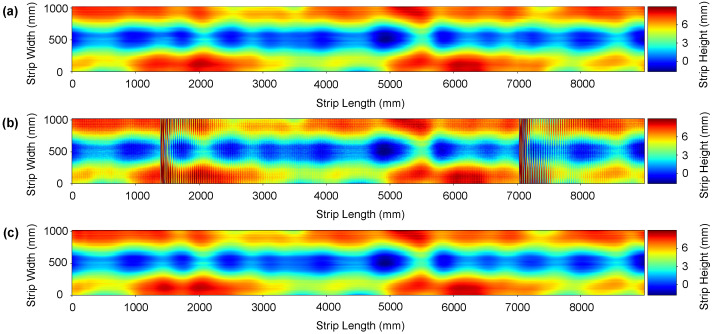
An instance of a real metal sheet surface denoising. Plate thickness: 3 mm; yield point Re: 215 MPa; Dimension of the mother plate—length: 9 m; width: 1050 mm. (**a**) Hermite filter denoised ground truth surface, (**b**) noise corrupted surface and (**c**) denoised reconstructed surface using the proposed network. (Color online).

**Figure 13:**
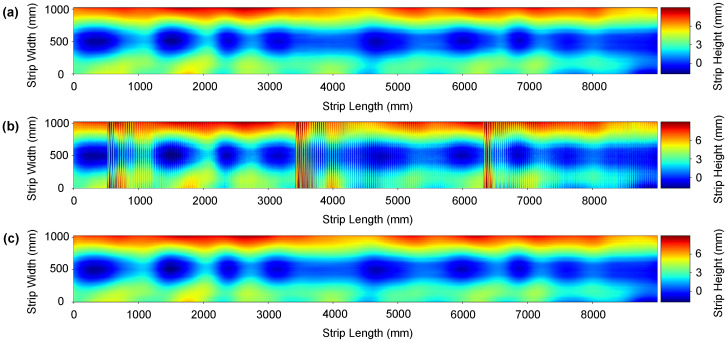
An instance of a real metal sheet surface denoising. Plate thickness: 6mm; yield point Re: 500 MPa; Dimension of the mother plate—length: 9 m; width: 1050 mm. (**a**) Hermite filter denoised ground truth surface, (**b**) noise corrupted surface and (**c**) denoised reconstructed surface using the proposed network. (Color online).

Measuring results from a specimen tested out of the roll levelling system with a CMM cannot be fairly compared to those obtained by our method, as has been previously discussed in Section 6. Results obtained with the double laser line sensor and the Hermite filtering method proposed by Alonso et al. [10] have been used as groundtruth in order to evaluate the improvement of the proposed method in an industrial environment. Experimental results with real data are shown in Figure 12 and Figure 13, where (a) is the denoised data using Hermite cubic interpolation, (b) is the raw data retrieved from the sensor and, finally, (c) is the denoised surface obtained using our method. The proposed CBRDNet architecture effectively recovers the smooth reconstructed surface after the noisy waves have been filtered, as seen in the figures.

**Figure 14:**
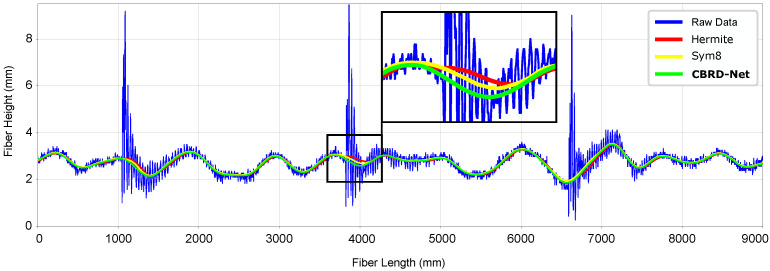
An instance of longitudinal fiber reconstruction, the blue line represents the raw 1D data from a fiber extracted from Figure 13, the red line shows the Hermite interpolation result, the yellow line shows Symlet results, and finally the green line depicts the CBRDNet denoised result. The inset provides a better detail of the results achieved by both Hermite and CBRDNet in the highlighted area.

The results shows graphically that the proposed method is capable of accurately reconstructing the surface of the metal sheet. When compared to state-of-the-art techniques, it achieves equivalent or better visually appealing results, as a real ground truth is always lacking in real experiments. Figure 14 depicts a longitudinal fibre, with unfiltered data collected directly from the sensor in blue, Hermite filtering in red, 2D Symlet wavelet-based filtering results in yellow, and the results from the CNN proposed in this work in green. It can be seen that the method is capable of reconstructing the sheet’s surface preserving the sinusoidal characteristics of the metal sheet, specially in areas where the cutting effect occurs.

### 7.3. Ablation Studies

Several ablation studies have been carried out in order to analyse the effects of both the noise estimation module (NE-SNet subnetowk) and training the network with synthetic, real, and mixed datasets.

#### 7.3.1. Effect of the NE-SNet Subnetwork

**Figure 15:**
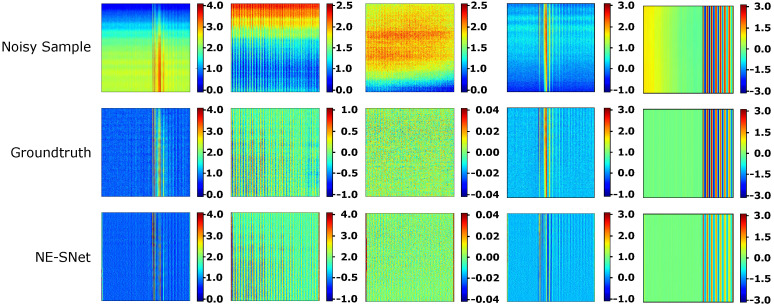
Results of the proposed NE-SNet subnetwork estimating the noise level map over a set of real and synthetic data samples. Noisy Sample stands for the corrupted noisy data, groundtruth is the known noise level map, finally NE-SNet is the estimated noise output. Color scale values are expressed in millimeters (mm). (Color online).

An ablation study was conducted to better understand the contribution of the NE-SNet subnetwork component to the overall system. This research has revealed that the overall performance of the proposed system is highly dependant on the NE-SNet subnetwork, increasing the accuracy of the proposed network up to 10%. Quantitative results of this study are shown in Table 3. Besides that, noise prediction experiments reveal that the NE-SNet achieves an accuracy of nearly a 85% extracting the noise both in synthetic and real data. Figure 15 depicts some results obtain by the NE-SNet subnetwok over both synthetic and real metal strip patches. The mean absolute error (MAE), maximum absolute error (MaxAE), standard deviation of the absolute error (STD), and root mean squared error (RMSE) were evaluated over a 500 sample dataset, results are as follows, MAE = 0.420 mm, MaxAE = 1.105 mm, STD = 0.124, and RMSE = 0.480 mm.

#### 7.3.2. Effect of Synthetic and Real Data

We have developed the following approaches. First, we trained our proposed CBRDNet on synthetic data exclusively. Second we trained CBRDNet on real data only. On the one hand, the experiments carried out demonstrate that CBRDNet (Synth) achieve worse results than CBRDNet (Real) and CBRDNet removing the existing real noise. This fact occurs even when trained on large amount of synthetic data samples, mainly because real noise cannot be accurately described by the defined noise model at Section 3. On the other hand, CBRDNet (Real) produces not so accurate results in comparison to CBRDNet, as a result of the impact of insufficiently noise-free real data. At the same time, CBRDNet has proved to be more effective in dealing with real noise while maintaining an accurate surface information. Quantitative results of the three strategies are shown in Table 4 on 500 sample synthetic, real, and mixed datasets. CBRDNet obtains better results than CBRDNet(Synth) and CBRDNet(Real) except in the synthetic dataset, but we dismiss these results as they are not directly applicable to a real production environment where real noise is present.

## 8. Conclusions and Future Work

In this paper, we present a novel denoising deep learning architecture for filtering range image sensor data that can be used for accurate flatness measurement in the context of metal sheet manufacturing, named CBRDNet.

This network is able to filter out the non-linear noise components in the range images that hinder accurate surface reconstruction and thus surface flatness measurements. It has been trained using both real and synthetic samples of steel coils from a roll leveling cut to length line. This combination improves the network’s denoising capabilities. Furthermore, synthetic data not only provided a wide range of representative samples for training, but also a groundtruth for quantitative evaluation of the accuracy of the denoised flatness measurements. We carried out different experiments to validate the proposed filtering strategy.

In the first place, results obtained denoising synthetic data have proved that our method outperforms traditional 1D filtering techniques, namely Hermite, Savitzky-Golay, Chebyshev, and Butterworth filters. Compared to them, we achieved an improvement of up to 6 times in terms of accuracy, particularly in surface regions where high amplitude noises are induced by the mechanical processes carried out in the production line, e.g., cutting the metal strip to the desired length. In the second place, the proposed CBRDNet achieves slightly better results in comparison with 2D wavelet-based filtering techniques. We achieved an error reduction up to 1.3 times when compared to the best performing wavelet in our study, i.e., Symlets (Sym8), although in some sample regions there was no clear improvement in terms of precision. Wavelet denoising results must be taken with a grain of salt, because an optimal wavelet class and order selection might improve them, while we report results of a necessarily limited empirical exploration. To this date we do not know of such a data driven optimal wavelet design process. In the third place, experiments with synthetic data show that the CBRDNet architecture is able to obtain better results than state-of-the-art deep learning denoising architectures for the specific kind of noise that we are dealing with. Compared to these methods we obtain improvements ranging from 1.2 up to 2.5 times in terms of surface reconstruction accuracy. This improvement is clearly visible in the areas of the metal sheet where the noise due to metal strip cutting occurs.

Finally, results with real data obtained from an industrial leveling cut to length line have shown that the proposed method is capable of accurately reconstructing metal sheet surfaces. The conducted experiments have shown a surface reconstruction error reduction than can be down to 15% relative to solutions based on conventional interpolation methods. Numerical results have shown that the proposed CBRDNet achieves a mean absolute error (MAE) of 0.140mm a maximum absolute error (MaxAE) of 0.376 mm, a standard deviation of the absolute error (STD) of 0.136 mm, and a root mean squared error (RMSE) of 0.147 mm.

Future research will explore deep denoising architectures in the frequency domain. Although in some cases it is difficult to differentiate a signal from noise in the spatial domain, this task might be easier in the frequency domain because noisy signals can be comprised of a set of sine wave signals represented in the frequency domain with different frequencies, phases, and amplitudes. We intend to implement and compare these possible enhancements to the network outlined in this paper in future works. Moreover, when larger data sets are needed but the access to real data is restricted in some way, for example, when data becomes sensitive to its distribution, or simply when access to real data is challenging, the development of tools capable of generating synthetic data would provide a solution to this data shortage. GANs are computational structures that employ two neural networks, competing with each other, to create new synthetic data samples that may be used as surrogates for real data. To further our research we plan to explore the potential of using GANs architectures instead of the current noise model to generate larger dataset with more likelihood to real data. 

## Figures and Tables

**Table 1:** Steel coils used for collecting real data and their specifications.

Coil	w × h (mm)	Young (GPa)	Poisson	Yield Stress (MPa)
S235JR	1050 × 3	205	0.301	215
S235JR	2000 × 8	205	0.301	215
S420ML	1650 × 7	190	0.290	410
S355M	1500 × 3	190	0.290	360
S500MC	1050 × 3	210	0.304	500
S500MC	1850 × 6	210	0.304	500

**Table 2:** Comparative results of our approach with both traditional 1D and 2D denoising approaches and 2D denoising CNN methods. MAE = mean absolute error; MaxAE = maximum absolute error; STD = standard deviation of the absolute error; RMSE = root mean squared error. Best results presented in bold font.

Method	CNN-2D/1D/2D	Blind/Non Blind	MAE *	MaxAE *	STD *	RMSE *
CBRDNet-ReLu (ours)	CNN-2D	Blind	**0.140**	**0.376**	**0.136**	**0.147**
CBRDNet-LeakyReLu (ours)	CNN-2D	Blind	0.160	0.466	0.154	0.172
CBDNet	CNN-2D	Blind	0.172	0.520	0.162	0.185
NERNet	CNN-2D	Blind	0.184	0.499	0.175	0.195
BRDNet	CNN-2D	Blind	0.198	0.659	0.184	0.212
FFDNet	CNN-2D	Non Blind	0.224	0.501	0.201	0.252
CDnCNN_B	CNN-2D	Blind	0.312	0.840	0.308	0.342
Sym8	2D	NA	0.176	0.543	0.170	0.188
Coif4	2D	NA	0.180	0.591	0.179	0.190
Db8	2D	NA	0.181	0.622	0.179	0.201
Dmey	2D	NA	0.256	0.942	0.282	0.291
Fk8	2D	NA	0.390	1.998	0.588	0.390
Hermite	1D	NA	0.413	1.150	0.380	0.459
Butterworth	1D	NA	0.760	4.423	0.735	0.781
Savitzky-Golay	1D	NA	0.842	6.436	0.779	0.853
Moving Average	1D	NA	0.801	5.463	0.928	0.865
Chebyshev Type II	1D	NA	0.828	5.040	0.828	0.903

* Measurements are expressed in millimeters (mm).

**Table 3:** Comparative results of our NE-SNet subnetwork ablation study with the full model and the best CNN and conventional denoising approaches. MAE = mean absolute error; MaxAE = maximum absolute error; STD = standard deviation of the absolute error; RMSE = root mean squared error. Best results presented in bold font.

Method	CNN-2D/1D/2D	MAE *	MaxAE *	STD *	RMSE *
CBRDNet (Full Model)	CNN-2D	**0.140**	**0.376**	**0.136**	**0.147**
CBRDNet (No NE-SNet)	CNN-2D	0.305	1.043	0.284	0.385
CBDNet	CNN-2D	0.172	0.520	0.162	0.185
Sym8	2D	0.176	0.543	0.170	0.188
Hermite	1D	0.413	1.150	0.380	0.459

* Measurements are expressed in millimeters (mm).

**Table 4:** Comparative results of training data ablation studies. MAE = mean absolute error; MaxAE = maximum absolute error; STD = standard deviation of the absolute error; RMSE = root mean. (Synth) = trained on synthetic dataset; (Real) = trained on real dataset squared error. Best results presented in bold font.

Method	MAE *	MaxAE *	STD *	RMSE *
**Mixed dataset results**				
CBRDNet	**0.140**	**0.376**	**0.136**	**0.147**
CBRDNet (Synth)	0.260	0.496	0.248	0.265
CBRDNet (Real)	0.180	0.401	0.175	0.186
**Synthetic dataset results**				
CBRDNet	0.190	0.410	0.181	0.195
CBRDNet (Synth)	**0.110**	**0.206**	**0.128**	**0.129**
CBRDNet (Real)	0.280	0.526	0.254	0.292
**Real dataset results**				
CBRDNet	**0.147**	**0.386**	**0.142**	**0.154**
CBRDNet (Synth)	0.282	0.366	0.265	0.291
CBRDNet (Real)	0.159	0.396	0.155	0.161

* Measurements are expressed in millimeters (mm).

## Abbreviations

The following abbreviations are used in this manuscript:

CNN	Convolutional Neural Network
DAE	Denoising Autoencoders
CDAE	Convolutional Denoising Autoencoders
GAN	Generative Adversarial Network
SGD	Stochastic Gradient Descent
CBRDNet	Convolutional Blind Residual Denoising Network
NE-SNet	Noise Estimation Subnetwork
NR-SNet	Noise Removal Subnetwork
ADAM	Adaptive Moment Estimation
ReLU	Rectified Linear Unit
BN	Batch Normalization
Conv2D	2D Convolution Layer
HSLA	High-Strength Low-Alloy
MSE	Mean Squared Error
MAE	Mean Absolute Error
MaxAE	Maximum Absolute Error
STD	Standard Deviation
RMSE	Root Mean Squared Error
CMM	Coordinate Measuring Machine
Db	Daubechies
Coif	Coiflets
Sym	Symlets
Fk	Fejer-Korovkin
Dmey	Meyer
